# Myocardial Regeneration via Progenitor Cell-Derived Exosomes

**DOI:** 10.1155/2017/7849851

**Published:** 2017-11-23

**Authors:** Janita A. Maring, Christien M. Beez, Volkmar Falk, Martina Seifert, Christof Stamm

**Affiliations:** ^1^Berlin-Brandenburg Center for Regenerative Therapies (BCRT), Charité-Universitätsmedizin Berlin, Corporate Member of Freie Universität Berlin, Humboldt-Universität zu Berlin, and Berlin Institute of Health, Berlin, Germany; ^2^German Center for Cardiovascular Research (DZHK), Partner Site Berlin, Berlin, Germany; ^3^Deutsches Herzzentrum Berlin (DHZB), Berlin, Germany

## Abstract

In the past 20 years, a variety of cell products has been evaluated in terms of their capacity to treat patients with acute myocardial infarction and chronic heart failure. Despite initial enthusiasm, therapeutic efficacy has overall been disappointing, and clinical application is costly and complex. Recently, a subset of small extracellular vesicles (EVs), commonly referred to as “exosomes,” was shown to confer cardioprotective and regenerative signals at a magnitude similar to that of their donor cells. The conceptual advantage is that they may be produced in industrial quantities and stored at the point-of-care for off-the-shelf application, ideally without eliciting a relevant recipient immune response or other adverse effects associated with viable cells. The body of evidence on beneficial exosome-mediated effects in animal models of heart diseases is rapidly growing. However, there is significant heterogeneity in terms of exosome source cells, isolation process, therapeutic dosage, and delivery mode. This review summarizes the current state of research on exosomes as experimental therapy of heart diseases and seeks to identify roadblocks that need to be overcome prior to clinical application.

## 1. Introduction

Regenerative therapy has the ultimate goal of repairing damaged tissue by limiting the extent of tissue damage following injury (cytoprotection), stimulating the endogenous regenerative capacity of a tissue (regeneration), and/or providing new cells or tissues as a replacement (cell therapy, tissue engineering). Although resident cardiac progenitor cells (CPC) have been described to persist within the adult mammalian myocardium [1–3], the myocardium has no clinical relevant intrinsic regenerative capacity due to a lack of postnatal cardiomyocyte mitosis [4]. Exogenous somatic cells transplanted into the diseased human heart failed to induce a meaningful improvement of heart function in clinical trials [5–8]. Cardiomyocyte cell products derived from embryonic or induced pluripotent stem cells (ES or iPS) have not been tested yet in controlled clinical trials, largely due to the complexity and costs of a clinical-grade production process. Concerns about genomic integrity of pluripotent-derived cells and unresolved issues such as cellular immaturity, coupling with host cells and possible arrhythmia, are difficult to rule out in suitable preclinical models.

Nevertheless, a variety of beneficial effects following experimental cell therapy in cardiac disease models has been observed even in the absence of transplanted cell persistence or stem/progenitor cell differentiation [9]. For instance, studies in which mesenchymal stromal cells (MSCs) were transplanted failed to show permanent engraftment of the transplanted cells but still yielded a sustained increase in cardiac function [10, 11]. Indeed, subsequent research has shown that conditioned medium collected from MSCs has cardioprotective effects and their secreted factors alone were already able to reduce infarct size and increase cardiac function in an animal myocardial infarction (MI) model [12, 13]. Investigations regarding the active factors secreted by the MSCs have shown that extracellular vesicles (EV) rather than single growth factors convey this beneficial effect [12].

Intercellular communication was long thought to be restricted to single agent messengers such as secreted growth factors and cytokines. Nowadays, the mode of paracrine signalling is extended by the incorporation of EVs as a major player in cell-to-cell as well as organ-to-organ communication. Vesicular structures were found in the extracellular space (and in body fluids) consisting of exosomes (30–100 nm), microvesicles (100–1000 nm), and apoptotic bodies (up to 5000 nm) [14]. Extracellular vesicle discovery is commonly attributed to the context of platelet maturation and the first use of the term dates back to the 1970s [15]. Extracellular vesicles are surrounded by a phospholipid membrane, and they are believed to contain up to 20,000 different protein molecules with preserved catalytic and ligand-binding activities [16–18]. More recently, vesicles were shown to also contain mRNAs and miRNAs, which seem to play an important role in cell-to-cell information transfer [19]. Extracellular vesicles are secreted in a diverse matter such as secretion via multivesicular bodies or pinching from the cell membrane. Exosomes are a distinct microvesicle subpopulation due to their particular biogenesis and the lack of proteins from certain organelles such as the Golgi apparatus and the endoplasmic reticulum, which can be found in other vesicles, for example, apoptotic bodies [20, 21].

Exosomes are formed through invaginations of endosomes, forming multivesicular bodies (MVBs) in the cytoplasm. Their content of RNAs, lipids, and proteins is partially a reflection of the composition of the cell. Therefore, part of the constituents found in exosomes is highly dependent on the state of the cell and changes upon, for example, hypoxia, mitochondrial stress, and differentiation [22]. Some components can be selectively sorted into the vesicles, for example, sorting of proteins into the exosomes is depended on the ESCRT (endosomal sorting complexes required for transport) machinery [23] or sphingolipids [24]. Interestingly, several studies have shown that the RNA content is not necessarily identical between donor cell and their secreted exosomes, since some microRNAs (miRNAs) are more abundantly present in vesicles compared to their originating cells, while others were completely absent [25–27].

**Figure 1:**
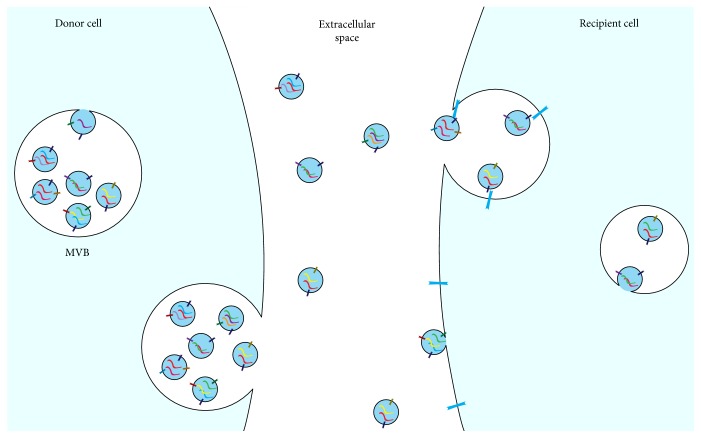
Exosomes are formed by invaginations of intercellular vesicles such as endosomes, which then form multivesicular bodies (MVBs). Exosomes are released into the extracellular space by fusion of the MVB with the cell membrane. Recipient cells take up the exosomes through direct fusion with the cell membrane, through internalisation or through receptor-ligand interaction on the recipient cell membrane.

Exosomes are secreted through transport and subsequent fusion of the MVBs to the cell membrane. This process is mediated by proteins of the Rab family, with Rab27A and Rab27B being the most prominent. Knockdown of either of these two proteins results in a significantly reduced exosome secretion [28, 29]. The process of exosome formation, secretion, and uptake known to date is shown in Figure 1. The bilipid membrane of the exosomes protects the content from degradation and thus enables signalling over long distances [19]. Investigations into the signalling range have revealed that the application of cells engineered to express Cre was able to rearrange the genome of susceptible cells containing a LoxP site on the contralateral site of the mice via their released EVs [30].

## 2. Suitable Cell Sources for Exosomal Therapy

**Figure 2:**
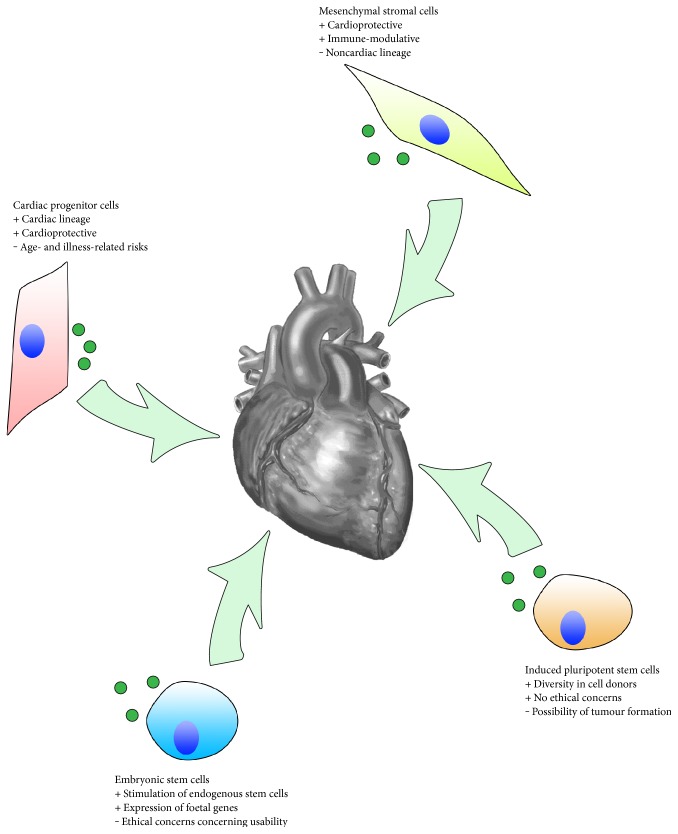
Several cell sources are being examined for use as an exosome therapy, most prominently cardiac progenitor cells, mesenchymal stem cells, and induced pluripotent stem cells as well as embryonic stem cells. Each of them has distinct advantages and disadvantages regarding cardiac regeneration.

Several cell sources could be used for a clinical approach with exosomes as a therapeutic agent. Multiple studies in several research areas have shown the effects of exosomes on, for instance, cell survival, angiogenesis, and migration [31, 32]. With the experience of the cells previously used in cell therapy, which were shown to be safe and effective, these donor cells are widely considered to be the most efficient sources for regenerative exosome generation (Figure 2).

The first cell type to be studied in preclinical and clinical settings for cardiac repair was bone marrow-derived cells, due to the fact that these cells are easily obtained in an autologous fashion. Even though it was hypothesized that bone marrow-derived cells were capable of transdifferentiating into cardiomyocytes [33], no conclusive evidence has been found that this is indeed the case [34, 35]. Although these cells were able to increase cardiac function when injected after myocardial infarction *in vivo* [33], they have varying results in clinical trials [5, 6].

More recently, MSCs have been investigated for clinical cardiac repair in autologous as well as allogeneic setting. In general, MSCs can be found in many tissues, such as bone marrow, adipose tissue, and cord blood. A hallmark is their capability to differentiate towards osteogenic, chondrogenic, and adipose lineages [36]. Differentiation into cardiomyocytes has so far only been seen in foetal MSCs, but with low efficiency *in vitro* [37]. The results on cardiac function in clinical trials have been very modest and not to the same extent as in the preclinical models [7, 8]. Moreover, MSCs have also been shown to not differentiate *in vivo* to cardiomyocytes and are not retained in the heart, suggesting that paracrine factors of these cells are the acting agents [10].

With the finding that the heart contains progenitor cells (CPC), actual cardiac regeneration seemed obtainable. Indeed, cardiac progenitors have the capability to differentiate into cardiomyocytes *in vitro* and *in vivo* [1–3, 38]. Moreover, they have been shown in preclinical models to be effective in increasing cardiac function after myocardial infarction [1–3, 38]. This concurred with the differentiation into cardiomyocytes and endothelial cells and, for example, an increase in angiogenesis in the borderzone of the infarction [9, 38]. Two clinical trials have been carried out so far to assess the safety of injecting cardiac progenitor cells. Both trials—CADUCEUS and SCIPIO—have indeed shown that injecting cardiac progenitor cells one month or more after myocardial infarction is safe, with some minor decreases in scar size and increases in local ejection fraction [39, 40].

Other cell sources are ES and iPS cell pluripotent stem cells, which have been under investigation for a wide range of regenerative processes since they still contain the ability to differentiate towards every cell type in the body. Therefore, pluripotent stem cells and cardiomyocytes derived from pluripotent stem cells have also been evaluated after myocardial infarction in preclinical models. In these studies, it was shown that these cells were indeed able to engraft into the heart and increase cardiac function significantly [41–44]. However, additional risks have emerged by using these cells, such as arrhythmias and teratoma formation [42–44].

## 3. Exosomes as Therapeutics in Cardiovascular Repair

Exosomes have been shown to be involved in a plethora of cellular processes, such as migration, differentiation, survival, and immune modulation [31, 32, 45]. Therefore, exosomes generated from the proper regenerative cell source could have profound beneficial effects in the regenerative processes after myocardial infarction, making them interesting new therapeutic agents. Due to the fact that the cells investigated in cell therapy after MI have been shown to convey their effects mainly through paracrine signalling, research has focused on evaluating the regenerative potential of the exosomes from these cell sources.  Table 1 provides an overview of the experimental studies performed so far, their major findings, as well as involved pathways or proposed active molecules.

One of the first studies evaluating the paracrine mechanism in cardiac repair used exosomes from human MSCs. Here, they showed that injection of MSC-derived exosomes into the tail vein of mice 5 minutes before reperfusion of the cardiac tissue significantly reduced infarct size 24 hours postoperation. Additionally, animals treated with exosomes had increased cardiac function compared to control animals over a 28-day time course. Analysis of the hearts showed that in the first 24 hours, there was an increase in activation of pAkt and pGSK3 (glycogen synthase kinase 3), which are both involved in cell survival pathways, whereas immune cell infiltration was decreased [32]. In another study, the cardioprotective effect of human MSCs was compared with their exosomes, both after injection into the infarcted borderzone. Both cells and exosomes comparably decreased infarct size after 28 days and increased cardiac function. Additionally, vessel density was also increased after the treatment with exosomes [46]. This proangiogenic effect of MSC exosomes has indeed been verified *in vitro* by these and other studies, where a significant induction of proliferation, tubule formation, and migration of endothelial cells was observed [46, 47]. This effect might be explained by the presence of several proangiogenic proteins in the exosomes, such as vascular endothelial growth factor (VEGF) and matrix metalloproteinase (MMP)-9 [47].

Exosomes from rat MSCs were shown to increase the proliferation, migration, and tubule formation of cardiac stem cells *in vitro*. Interestingly, it was demonstrated that several miRNAs were changed into these cells upon exosome treatment, such as upregulation of miR-147 and miR-503-3p and downregulation of miR-207, miR-326-5p, and miR-702-5p [48]. When cardiac stem cells were treated with exosomes from rat MSCs prior to injection into the heart, an increase in cardiac function and vessel density at the infarct site could be seen after 28 days [48].

Exosomes from iPS or ES cells were also able to increase proliferation and tubule formation in CPCs [49], while apoptosis was reduced *in vitro* in both CPCs and cardiomyocytes [49, 50]. Furthermore, the expression of several endothelial and cardiomyocyte genes was upregulated in CPCs after stimulation with ES-derived exosomes. When injected into the heart after MI, the exosomes were able to reduced apoptosis after 48 hours [50] and 4 weeks [49]. Furthermore, injection of ES-derived exosomes increased proliferation, vessel density, and cardiac function, while injection of CPCs prestimulated with ES-derived exosomes, increased cardiac function, and reduced infarct size. The cardioprotective effect could be due to the presence of several known protective miRNAs present in the exosomes, such as miR-21 and miR-210 [50]. The importance of miR-291, miR-294, and miR-295 was confirmed when the use of a miRNA mimic was able to reproduce the induction of CPC proliferation *in vitro* [49].

CPC-derived exosomes are currently being investigated regarding their regenerative potential. Mice CPC-derived exosomes have been shown to reduce the apoptosis of cardiomyocytes *in vitro* and *in vivo* after MI [51]. Exosomes from rat CPCs were able to increase tubule formation of endothelial cells. Furthermore, the mRNA levels of several fibrosis-related genes were significantly downregulated. *In vivo* analysis of these rat CPC-derived exosomes showed an increase in cardiac function, while fibrosis was reduced [52]. Also, exosomes from human CPCs have been evaluated whether they are able to reproduce the positive effects of the originating cell type when injected after MI. Those CPC-derived exosomes were capable to reduce the infarct size in between 7 and 30 days after MI, accompanied by an increase in cardiac function and vessel density. Furthermore, fibrosis was markedly reduced [53, 54]. It became apparent that miR-146a seems to be an important mediator in those exosomes, as a mimic of the miRNA was able to partly reproduce the antiapoptotic effect of the exosomes *in vitro*. Further investigations regarding the role of miR-146a *in vivo* by a knockout mice model showed that without miR-146a infarct size was not reduced and cardiac function did not improve, while injecting a mimic or miR-146a after MI did [54]. The overall proangiogenic effect of CPC-derived exosomes has been implicated in the abovementioned studies. Indeed, exosomes from CPCs are able to promote several parameters of the angiogenesis process *in vitro*. Endothelial cells stimulated with human CPC-derived exosomes markedly increase migration, tubule formation, and spheroid sprouting [31, 47]. Moreover, in a mouse matrigel-based plug assay, overall cell migration into the plug and blood vessel formation was increased in the presence of exosomes. Several proangiogenic factors were present in these exosomes, such as VEGF, MMP-9, and EMMPRIN (extracellular matrix metalloproteinase inducer). Knockdown of EMMPRIN showed a significant decrease in angiogenesis *in vitro* and *in vivo*, implying that EMMPRIN is a major factor in CPC-derived exosome-induced angiogenesis [47].

## 4. Potential of Modified Exosomes

Since the protein and miRNA content of exosomes depended on the state and content of their donor cells, several approaches are possible to increase the efficiency of exosomes on cardiac repair. CD34^+^ cells were genetically modified to overexpress sonic hedgehog (SHH), which resulted in an increased cardiac function and vessel density after MI. It was found that this was (partly) due to the fact that the exosomes from these cells contain SHH, which can be transferred to the recipient cells within the heart, and increase SHH signalling [55].

Overexpression of GATA binding protein 4 (GATA4) in rat bone marrow-derived MSCs leads to a reduction of hypoxia-induced apoptosis of cardiomyocytes when treated with the exosomes from these cells. Moreover, GATA4-overexpressing exosomes restored mitochondrial integrity and had an increase in miR-19 content. MiR-19 is an important effector in survival pathways. *In vivo* analysis indeed showed an increase in miR-19 when the GATA4-overexpressing exosomes were injected after MI. This also leads to an increase in cardiac function and a reduction in infarct size [56].

Besides genetically altering the donor cell to increase or decrease the expression of certain proteins, exosomes can also be altered by changing the conditions of the donor cell at the time of exosome generation. For example, hypoxia is well known to have profound effects on cells, and (remote) ischemic preconditioning has been shown to have positive effects in the clinic [57]. When exosomes were isolated from the hearts or MSCs that have undergone ischemic preconditioning, these exosomes reduced infarct size and fibrosis after MI [58, 59]. *In vitro*, they were able to reduce apoptosis in cardiomyocytes [58].

## 5. Translation to Preclinical Large Animal Models

Recently, the Marban group reported on a series of experiments in a clinical relevant pig model, where exosomes secreted by human cardiosphere-derived cells were delivered either intracoronary or intramyocardial following reperfused myocardial infarction [60]. In the acute infarct model, only intramyocardial exosome injection resulted in reserved LV function. Moreover, intramyocardial injection was also beneficial in chronic ischemia. The obvious conclusion is that exosomes need to be delivered directly into the myocardium, which may render clinical application even more complex. However, cohort size was small, and the average particle diameter was 197 nm rather than the typical diameter size of 30–100 nm. The authors attribute this to their use of nanoparticle tracking analysis, but it cannot be ruled out that this reflects a different vesicle population [60]. The notion that exosomes are poorly taken up after intracoronary delivery indeed needs serious consideration. Within tissue, cell-to-cell transfer of exosomes between neighbouring cells is feasible. For adhesion to and uptake by the vascular endothelium, however, a minimum density and number of specific adhesion molecules presented on the cell surface are required. On nanoscale exosomes formed in MVBs, a small number of surface proteins may be randomly incorporated, and exosomes should transit the capillary bed at a higher speed than the entire cell. It remains to be investigated whether the exosomes secreted from stimulated cells with distinct endothelial adhesion are predisposed to be taken up by the targeted cells. Interestingly, the Marban group also showed in acute ischemia-reperfusion experiments in rats and mice that CPC-derived exosomes confer cardioprotection by modulating macrophage polarization, induced by transfer of miR-181b leading to inhibition of protein kinase C (PKC)*δ* [61]. Interestingly, these effects were observed after intracoronary infusion of the exosomes in a rat model and after intramuscular delivery in pigs [60, 61]. In another study, the same group implicated that the Y RNA fragment (EV-YF1) is responsible for cardiomyocyte protection form hypoxia-reoxygenation/ischemia-reperfusion injury by being transferred to macrophages and inducing IL-10 secretion [62]. The means by which exosomes modulate the local tissue immune balance via small RNAs is clearly emerging. Individual pathways and mediators can be identified in specific experimental models, but there is consensus that the multitude of exosome nucleic acids (and proteins) acting in concert are responsible for their pronounced and sustained cardioprotective effects.

## 6. Future Perspectives

Exosomes from sources such as MSCs or CPCs have been investigated as interesting new therapeutical agents. So far, the first preclinical studies have shown that these exosomes have indeed large regenerative potential and are able to positively influence important processes after myocardial infarction. These effects were observed on a cellular as well as on the whole organ as an overall improvement in cardiac function.

### 6.1. Clinical Translation and Commercial Exploitation

A number of companies have a secured intellectual property of exosome technology, such as Esperite (immunology applications), Aegle Therapeutics (burns, chronic wounds, etc.), ReNeuron (neural stem cells), SystemsBiosciences (extracellular vesicle precipitation), Anosys (dendritic cell-derived ECV for malignancies), and Capricor. Capricor is a Cedars Sinai Medical Center Spin-off run by the Marban family that developed and evaluated cardiosphere-derived progenitor cells for treatment of postischemic myocardial dysfunction. Their allogeneic CPC product, CAP-1002, apparently did not meet the efficacy primary outcome in a controlled clinical trial, and the company now focuses on clinical translational of their CPC-derived exosome product (CAP-2003). Their claim, that only exosomes derived from CPCs are effective in ischemic heart disease, is supported by data published by the Marban research group, [54, 60, 62]. However, other groups showed that exosomes from other cell sources are cardioprotective, too [32, 63].

### 6.2. Exosomes and Immunology

Exosomes derived from dendritic cells or B-cells were shown to present functional surface MHC class I and II antigens [17, 18]. This phenomenon is the basis for the concept of using exosomes/vesicles designed to contain specific tumour antigens as tumour vaccines. Similarly, vesicles carrying antigens of infectious pathogens may be used to elicit a specific immune response for vaccination or treatment. The current assumption is that exosomes from nonimmunological cells are immune-privileged, and indeed most animal experiments so far have been performed with exosomes derived from allogeneic or xenogeneic cells. Even if transplanted exosomes are ultimately eliminated by phagocytic cells, this does not seem to influence their therapeutic capacity. In contrast, in a recent study from Sicco et al., the immune-modulating property of exosomes from MSCs was investigated regarding their effects on macrophages. Here, they could show *in vitro* that a switch towards alternative activated macrophages can be induced by MSC-derived exosomes, which might explain to a certain extent their therapeutic potential [64]. Whether an unwanted sensitization can be induced by exosome-related alloantigens or allogeneic exosomes are less effective in allosensitized patients is not known, yet, but needs further attention.

### 6.3. Autologous Exosomes

In principle, exosomes can be obtained from autologous cells, provided that the primary cell source possesses robust viability and proliferation capacity in cell culture in order to yield sufficient high cell numbers and, if needed, responds to stimulation for collection of large quantities of conditioned media. MSCs, fibroblasts, endothelial cells, and also cells of cardiac origin (e.g., CPC) generally meet these criteria, but cultivation may be difficult or fail in patients of advanced age or severe disease, as well as a result in less effective exosomes [65]. However, the process of obtaining therapeutic doses of autologous exosomes could be time-consuming and costly, and their application in emergency situations such as acute myocardial infarction is not feasible.

### 6.4. Exosomes as a Therapeutic Product

While exosomes are not organisms, they do contain a nonuniform mix of proteins and are derived from cells that are either allogeneic or autologous but subjected to nonhomologous use. Therefore, they meet the key criteria for classification as biological medicinal products or, if derived from manipulated cells, as advanced therapeutic medicinal product (ATMP) or gene therapy products by most authorities. An algorithm for regulatory classification of extracellular vesicles has recently been proposed in a position paper of the International Society for Extracellular Vesicles (ISEV) [66]. As outlined by Brindley et al., the characterization of clinical-grade exosomes and their cGMP-compatible production process should encompass a defined size range including the use of a standardized detection method(s), identification by defined biochemical markers, exosome purity (freedom from cells, cell debris, and macromolecules), scalable isolation methods including serum-free source cell culture, and methods for exosome stabilization and storage [67]. It has been suggested that exosomes may be commercialized as “by-products” of cell manufacturing, but we consider it likely that dedicated exosome production processes will be required. Several groups concentrate on using iPS-derived cells as donor cells to generate exosomes for therapeutic use, such as iPS-derived MSCs. The idea is to use a standardized, “perfect” cell source free from any pathogens of genetic aberrations, possibly customized to enhance exosome function, to yield a uniform exosome product that is “streamlined” in terms of compliance with regulatory demands.

### 6.5. Optimizing Exosomes

In addition to their intrinsic therapeutic potential, exosomes may be used as vehicles for delivery of small molecule drugs, proteins, and nucleic acids (comprehensively reviewed by Ha et al.) [68]. This may be particularly useful when the active compound is unstable, such as siRNA. Moreover, exosomes may enable drugs to enter the central nervous system that are not able to cross the blood-brain barrier in the “naked” form. Specific delivery of cytostatic drugs to tumour tissue via integration into exosomes has also been described [69]. Compared to artificial liposomes for drug delivery, exosomes are believed to persist longer and to possess additional biologic effects [70].

To enhance therapeutic efficacy in the heart, it has been suggested to harvest exosomes from cells that were stimulated by stressors such as hypoxia, leading to an accumulation of stress-response proteins and possibly nucleic acids. While exosomes contain integrins, not all adhesion molecules present on exosomes have been identified yet. This might be the reason that not all exosomes are effective upon systemic or intracoronary delivery, as was shown by the Marban group in a large animal myocardial infarction model [60]. However, in numerous rodent models, intravascular exosome delivery led to improved cardiac function and it is entirely unclear whether this phenomenon has specific biologic reasons or merely reflects local underdosing [32, 71]. Guiding exosomes to specific tissues and improving their uptake by manipulating their surface protein profile are a field of research that will be important for therapeutic success.

## 7. Conclusion

Extracellular vesicles, most notably exosomes, have been known for almost 50 years, but their potential for therapeutic use in regenerative medicine has only recently been acknowledged. Numerous basic research and preclinical studies have shown beneficial effects in models of different heart diseases, including the acutely or chronically ischemic heart. Compared to viable cell products, development of a readily available off-the-self therapy should be less complex. However, as was the case with “whole cell” therapy for heart disease in the past 20 years, exosomes from easily obtainable unmodified cells may have limited therapeutic efficacy, and future clinical studies will soon clarify this. Many facets of current translational and commercial activities surrounding the use of exosomes in cardiac regeneration are evocative of the recent history of somatic cell therapy. It remains to be seen whether exosome biology indeed offers fundamental advantages and is able to make a relevant contribution to the development of novel therapies for heart disease.

## Figures and Tables

**Table 1:** Overview of exosomes as therapeutics in preclinical MI studies.

Cell type	Species	Donor cell specifics	Cardiac effect					Other effects	Active pathways/molecules	Ref
			Diminished infarct size	Increased cardiac function	Higher vessel density	Reduced fibrosis	Decreased apoptosis			
MSC	Human		✓	✓	✓					[46]
	Human		✓	✓						[32]
	Rat	CPCs prestimulated with exosomes		✓	✓	✓				[48]
	Mouse	Ischemic preconditioning				✓		Increase in pAkt and pGsk3, reduced immune cell infiltration	miR-22	[58]

CPC	Human	Cardiac explant cells from cardiovascular patients		Reduced				Decrease in exosome production	IL-6	[65]
	Human		✓	✓	✓	✓	✓		miR-132	[53]
	Human		✓	✓	✓	✓			miR-146a	[54]
	Rat			✓		✓			miR-292	[52]
	Mouse						✓		miR-451	[51]

ES	Mouse		✓	✓	✓		✓	Increase in CPC proliferation	miR-291, miR-294, miR-295/Akt	[49]

iPS	Mouse						✓		miR-21, miR-210	[50]

Other	Human	hCD34+ SHH+						Transfer of SHH to endothelial cells	SHH	[55]

	Rat/human	Plasma from remote ischemic preconditioning	✓						Erk, Akt	[71]
	Rat	Perfused hearts with ischaemia (conditioning)	✓							[59]
